# Comparing Leishman and Giemsa staining for the assessment of peripheral blood smear preparations in a malaria-endemic region in India

**DOI:** 10.1186/1475-2875-13-512

**Published:** 2014-12-30

**Authors:** Sanghamitra Sathpathi, Akshaya K Mohanty, Parthasarathi Satpathi, Saroj K Mishra, Prativa K Behera, Goutam Patel, Arjen M Dondorp

**Affiliations:** ‘Anusandhan’ Research Laboratory, Ispat General Hospital, Rourkela, Odisha India; Department of Microbiology, Midnapore Medical College and Hospital, West-Bengal, India; Faculty of Tropical Medicine, Mahidol University, Bangkok, Thailand; Centre for Tropical Medicine, Nuffield Department of Medicine, University of Oxford, Oxford, UK

## Abstract

**Background:**

Microscopy of peripheral blood thin and thick films remains the reference for malaria diagnosis. Although Giemsa staining is most commonly used, the Leishman staining method provides better visualization of the nuclear chromatin pattern of cells. It is less well known whether accuracy of parasitaemia assessment is equally accurate with the latter method.

**Methods:**

Peripheral blood thin and thick smears from consecutive febrile patients admitted to Ispat General hospital, Rourkela, Odhisa, India, were stained with Giemsa and Leishman stain. Methods were compared for species identification, parasite quantification, and ability for identification of alternative diagnoses.

**Results:**

Blood films from 1,180 fever patients were compared according to staining method, of which 111 were identified as parasitaemic using Giemsa and 110 with Leishman staining. The Kappa value as a measure of agreement between methods was 0.995 (p < 0.001), and the log_10_parasitaemia between methods were strongly correlated (r^2^ = 0.9981). In parasite negative patients, thin smear assessment contributed to making a diagnosis in 276/1,180 (23%) of cases. These assessments were better made in Leishman-stained preparations, especially for the assessment of morphological changes in red and white cells.

**Conclusion:**

Leishman’s staining method for thin and thick smears is a good alternative to Giemsa’s stain for identifying *Plasmodium* parasites. The Leishman method is superior for visualization of red and white blood cell morphology.

## Background

Early parasitological diagnosis of malaria is the cornerstone of malaria treatment and control. Although rapid diagnostic tests for malaria are increasingly used, microscopy remains the reference standard for malaria diagnosis [1]. Microscopy has the advantage of providing a quantitative assessment of peripheral blood parasitaemia and parasite stages, as well as information on the other blood elements [2]. Sensitivity of thick smear evaluation exceeds 80% at a parasitaemia above ten parasites per μL [3]. Depending on the techniques used, real-time polymerase chain reaction (RT-PCR), is 50 times more sensitive than microscopy but the technique is expensive and requires a high level of technical expertise [4]. An accurate microscopic diagnosis requires a high quality smear and to achieve this proper staining of the smear is mandatory. Commonly used stains are aqueous Romanowsky, such as Field’s and JSB stain, or alcohol-based Romanowsky, such as Giemsa, Leishman and Wright stains [5]. Aqueous Romanowsky stains are commonly preferred in field settings in which there is a risk of evaporation of alcohol-based stains. These stains are also suitable for staining thick blood smears. Alcohol-based stains, such as Giemsa or Leishman, are suitable for both thin and thick smears and are most commonly used in better equipped laboratories with availability of well trained personnel.

In malaria-endemic regions, thin and thick smear preparation are used to diagnose malaria, but examination of the blood elements can also reveal other causes of fever including leukaemia, or clues for viral infection or bacterial sepsis. For this purpose, Leishman stain could be preferable to Giemsa, since visualization of the nuclear chromatin pattern and cytoplasmic colour contrast are known to be clearer with the Leishman method. The Leishman stain also takes less time for preparation than Giemsa staining. However, these advantages should not be at the expense of accuracy in parasitaemia assessment, which is the primary purpose of the malaria blood slide examination. In the current study the qualitative and quantitative assessment of *Plasmodium* parasites in Leishman compared to Giemsa-stained thin and thick peripheral blood films was assessed in patients presenting with fever at the time of admission in the hospital. The staining methods were also compared, regarding their contribution to suggesting alternative diagnoses than malaria.

## Methods

The study was performed at Ispat General Hospital (IGH), Rourkela situated in the Sundergarh district of Odisha, India. With a population of 36.7 million (3.5% of India’s total population), Odisha has the highest population at risk for malaria in India. In 2010 Odisha contributed nearly 24% of total cases and 17% of total malaria mortality in India [6]. Above 90% of malaria-attributable deaths in India are caused by *Plasmodium falciparum* [6]. IGH is a 685-bedded tertiary care hospital with a large catchment area beyond Odisha, including Jharkhand, Chhatisgarh and Madhya Pradesh. Malaria is seasonal with a peak incidence from September to December, although cases present throughout the year. In the present study, 1,180 fever cases admitted to IGH from August to December 2012 received a peripheral blood slide examination.

In the current study, EDTA anti-coagulated blood samples were collected from consecutive febrile patients admitted to the hospital. Samples were processed without delay to avoid morphological alteration of parasites related to storage [7]. A sample of 10 μL EDTA blood was then used for the simultaneous preparation of two thin and thick smear slides, one stained according to Giemsa and the other according to Leishman's method. Fixation of the thin smear was done in a covered staining jar containing anhydrous methanol for 1 to 2 min, after which the slides were air-dried. Giemsa working solution (Qualigens, product no 39382, batch no NL 06766308S) was diluted 1:15 with phosphate buffered water (pH7.2). Leishman working solution (S DFine-Chem Ltd, Product no 44042, batch no Lo4x/0504/0212/71) was diluted 1:6 using the same buffer. Every day a fresh filtered working solution was used. The slides containing the thick and thin smear were then submersed in either Giemsa staining solution for 30 min or Leishman stain for 12–15 min. Finally, slides were washed gently under running tap water and air dried prior to microscopic assessment.

Slides were independently read by two of the authors (SS and AKM). Both readers have more than 15 years experience in slide assessments. Assessments of presence of malaria parasites, quantification of parasites, and non-quantitative assessment of alternative diagnoses were compared between readers. In case of non-congruent results in the qualitative assessment of malaria species or in suggestions of alternative diagnoses, a consensus was reached through joint review of the case. In both Giemsa- and Leishman-stained slides, the thick smear was used for diagnosis and quantitative assessment, whereas the thin smear for species identification. Parasitaemia in all malaria- positive cases was quantified per 200 white blood cells (WBC), independently by two microscopists. Parasitaemia (PRST) per μL was calculated as: PRST/μL = 40 × PRST/200 WBC, assuming a WBC count of 8,000/μL. In case of high parasitaemia (>50-70 parasites/field in thick smear) a pinhole eyepiece was used to avoid over-estimation of parasitaemia.The microscopists were blinded to the status of the patient and the reading results of the alternatively stained slide from the same patient.

### Statistical analysis

For comparison of the quantitative parasite counts, the log_10_of the parasitaemia per μL was used to account for the natural increase in variability with increasing parasite density [8]. The correlation in log_10_ parasitaemia between methods was assessed by the method of Pearson. A Bland-Altman plot was produced comparing the differences between methods according to parasitaemia. Agreement of methods was assessed by calculation of the Kappa value and the intraclass correlation coefficient was calculated using SPSS statistical software package (IBM, SPSS Statistics version 20)

## Results

**Figure 1:**
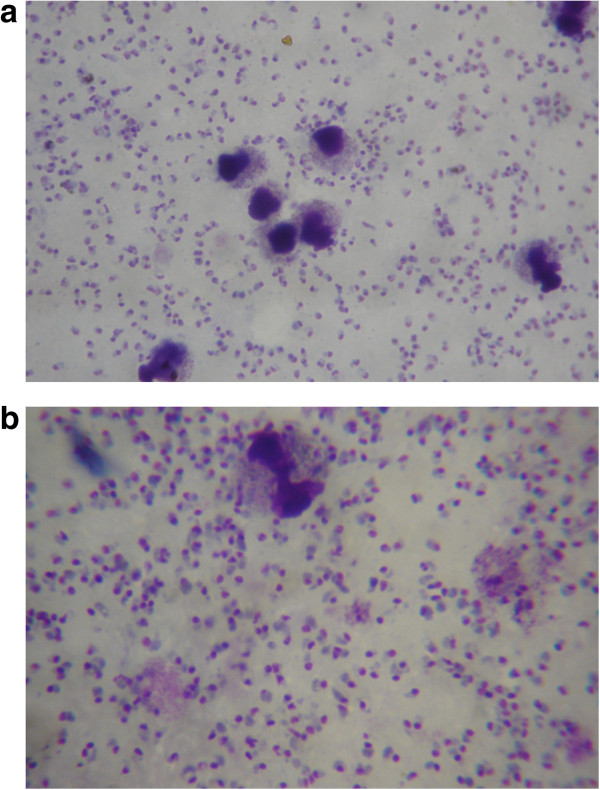
**Leishman (a) and Giemsa (b) stained peripheral blood thick smear showing ring stage** ***Plasmodiumfalciparum*** **.** Parasites are easier identified in the Giemsa-stained slides.

**Figure 2:**
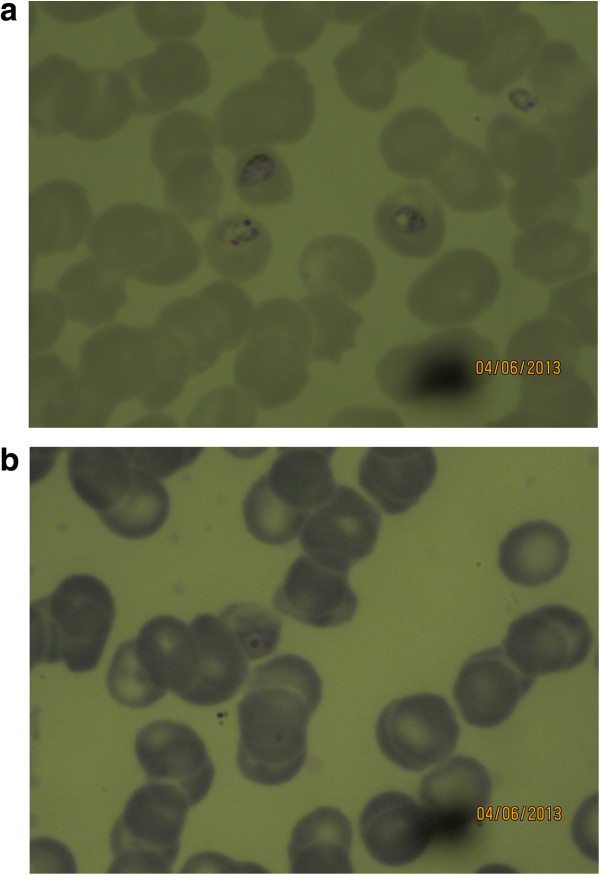
**Leishman (a) and Giemsa (b) stained thin smear showing mature trophozoite stage** ***Plasmodium falciparum*** **.** The contrast between parasite cytoplasm and red cell cytoplasm is more pronounced.

A peripheral blood film was taken from 1,180 patients with fever, and stained according to both Giemsa and Leishman. A total of 111 patients were parasitaemic by light microscopic assessment of the Giemsa-stained slide, compared to 110 patients with Leishman-stained slides. The Kappa value as a measure of agreement between methods was 0.995 (p < 0.001). A total of 276 patients showed other abnormalities (Table 1). According to two independent slide readers, subjective comparison of the methods revealed that parasite identification was easier in thick smear preparation stained according to Giemsa (Figure 1). However, in the thin smears, slides stained according to Leishman had a better colour contrast between the parasite cytoplasm and the red cell cytoplasm, in particular in case of mature trophozoite stage parasites (Figure 2).

**Table 1:** **Light microscopic blood slide findings in 1,180 fever patients examined at Ispat General Hospital, Rourkela, Odhisa, India**

No of cases	Diagnosis
111	Malaria (Pf = 92, Pv = 2, Pf + Pv = 17)
202	Shift to left with toxic granulation of neutrophils
68	Atypical lymphocytes
5	Suggestive of infectious mononucleosis
1	Acute lymphoblastic leukemia (ALL)
793	No cause of fever could be determined

**Figure 3:**
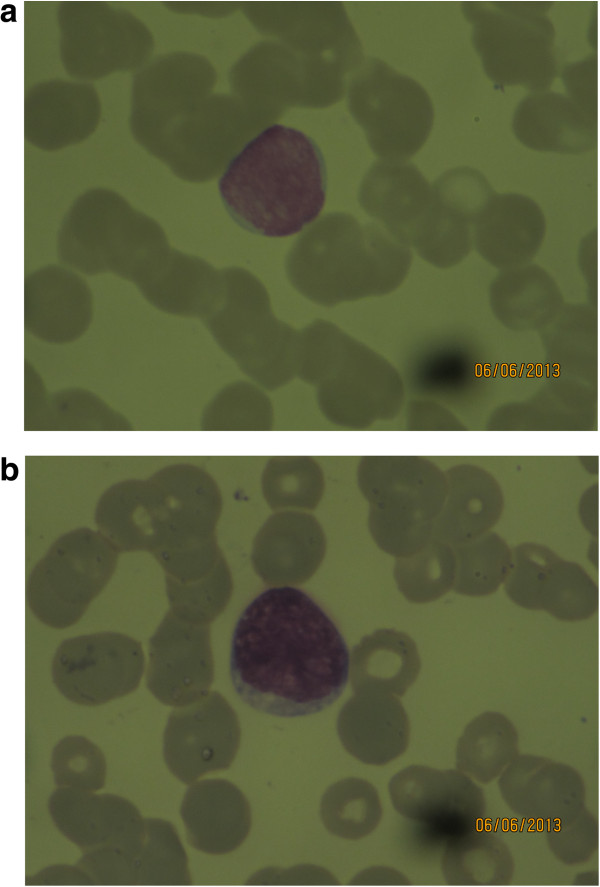
**Leishman (a) compared to Giemsa (b) stained peripheral blood slide showing a lymphoblast in a patient with acute lymphoblastic leukaemia (ALL).** In the Leishman-stained preparation the fine granular chromatin structure is better defined than in the Giemsa-stained preparation.

In parasite negative patients, thin smear assessment suggested an alternative diagnosis in another 276/1,180 (23%) of cases (Table 1). These additional findings were generally better shown in Leishman-stained preparations, compared to Giemsa- stained, especially for assessment of morphological changes in leucocytes. In patients with leukaemia, details of the nuclear chromatin and cytoplasm of the lymphoblasts were better appreciated with Leishman stain, which is a key feature for morphological categorization of leukaemia (Figure 3). Also, in non-leukaemia cases, the nuclear chromatin distribution and cytoplasmic granule colour and character were in general more clear in Leishman-stained preparations (Table 1, Figures 2 and 3).

**Figure 4:**
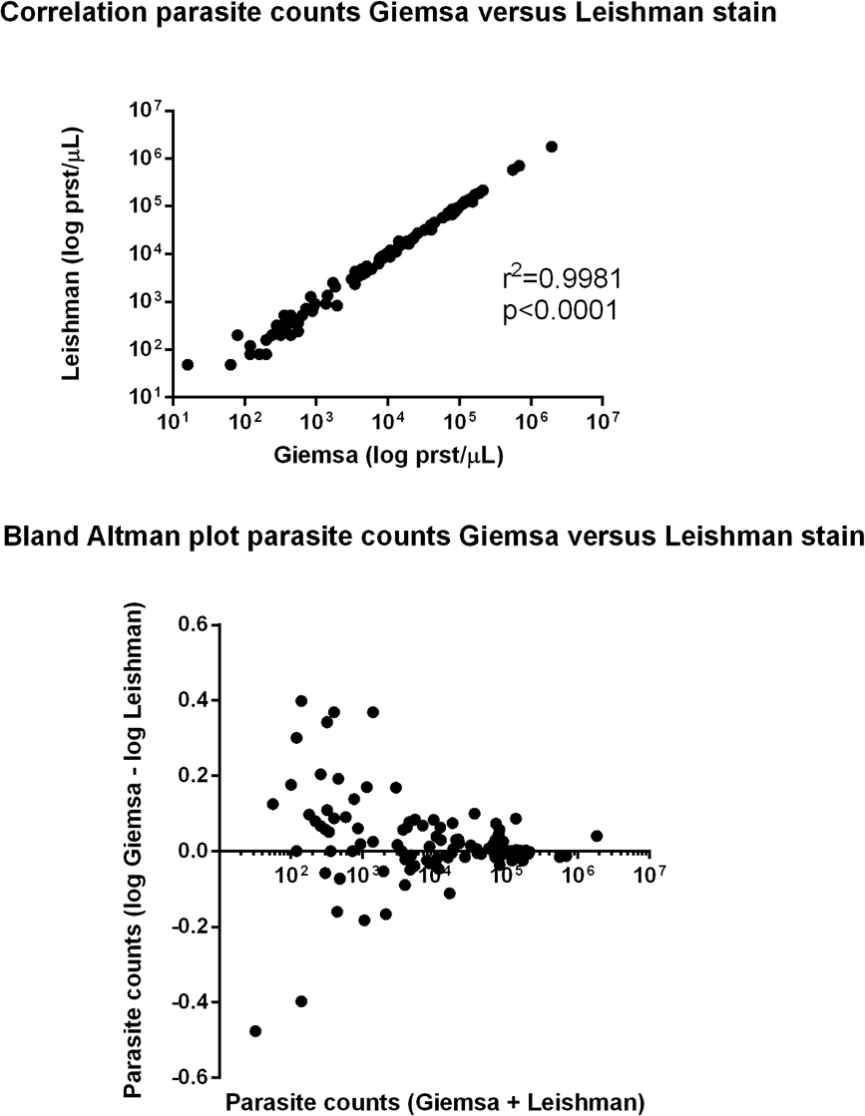
**Correlation of parasite counts and Bland-Altman plots comparing Giemsa with Leishman-stained thick films for quantification of** ***Plasmodium falciparum.***

The median quantitative parasite counts were 236 (range 0 to 19,39,264) PRST/μL in the Giemsa-stained slides and 281 (range 0 to17,68,448) PRST/μL in the Leishman-stained slides. Log_10_ parasitaemia between the two staining methods was strongly correlated (r^2^ = 0.9981; p < 0.0001, Figure 4). The difference in parasitaemia between methods increased with increased parasite density , but the reverse was true for the log transformed values (Table 2, Figure 4).

**Table 2:** **Absolute differences in parasite counts between Giemsa- and Leishman-stained blood slides**

Absolute difference in parasite count between methods	Mean difference	No of cases (%)
0-400	129	46 (41.44%)
401-800	587	19 (17.11%)
801-1,200	984	15 (13.51%)
>1,200	6,294	31(27.92%)
Total	1,687	111(100%)

## Discussion

This study shows that the Leishman-staining method is a good alternative to the Giemsa method for quantification of *Plsmodium falciparum* and *Plasmodium vivax* parasitaemia, and has the advantage that abnormalities in other blood elements, in particular WBC, are better identified. Parasite counts compared between the two separate methods were highly correlated and the methods showed a high level of agreement. As expected there was an increase in the absolute difference in parasite counts at a higher parasitaemia [8, 9]. Species and parasite asexual stage identification could be identified properly from the thin smear stained according to Leishman, and this was subjectively easier than in the Giemsa-stained slides. In falciparum malaria, the more mature trophozoite and schizont stages sequester in the microcirculation of vital organs, compromising microcirculatory flow and organ function. A preponderance of these more mature parasite forms in the peripheral blood is thought to represent a larger sequestered parasite burden and this is associated with more severe disease and poor disease outcome [10]. Assessment of these late-stage parasites was easier in Leishman-stained slides compared to Giemsa. Red cells stained with Giemsa result in a more bluish tinge than with Leishman stain. This makes the contrast of the parasitic cytoplasm with Giemsa less appreciable. In the thick smear preparation Giemsa stain was subjectively superior to Leishman. However, this did not translate into reduced sensitivity or reduced accuracy in quantitation in the series: only one case was missed with Leishman stain, and in general thick smear reading was not more difficult in preparations stained with Leishman.

Examination of the thin film enables additional diagnoses. In the series one case of acute lymphoblastic-leukaemia, five cases suggestive of infectious mononucleosis, 68 cases suggestive of viral infections based on the presence of atypical lymphocytes, and 202 cases of probable invasive bacterial infections (left shift and toxic granulation in neutrophils) were identified. In all these cases Leishman stain was superior to Giemsa stain for the morphologic assessment of leukocytes. The fineness of chromatin pattern and colour of the nucleus was clearer with Leishman stain compared to Giemsa. Also red blood cell morphology was more easily assessed with Leishman stain. In addition to this, the time taken for staining is shorter with the Leishman method, which can be an important advantage in countries such as India where diagnostic laboratories are sometimes understaffed.

Since light microscopy of the Giemsa-stained slides were used as the reference method, the study did not evaluate the sensitivity of the two staining methods for detection of low parasitaemias. Also, the comparison of preservation of the staining quality could be subject to future study.

## Conclusion

This study shows that the Leishman’s staining method for thin and thick smears is a good alternative to Giemsa for identifying *Plasmodium* parasites. The Leishman method is superior for visualization of red and white blood cell morphology, which can be an advantage for the diagnosis of diseases other than malaria identifiable by changes in these blood elements.
